# Mechanical Properties, Durability Performance, and Microstructure of CaO-Fly Ash Solidified Sludge from Northeast, China

**DOI:** 10.3390/ma17194757

**Published:** 2024-09-27

**Authors:** Chen Chen, Kai Zhang, Chunyu Ma, Zhigang Yin, Liang Wang, Yao Chen, Ziqi Lin, Yi Liu

**Affiliations:** 1 School of Civil Engineering, Institute of Disaster Prevention, Sanhe 065201, China; chenchen@cidp.edu.cn; 2 Key Laboratory of Building Collapse Mechanism and Disaster Prevention, China Earthquake Administration, Sanhe 065201, China; 3 Research Center of Earthquake Engineering, China Institute of Water Resources and Hydropower Research, Beijing 100048, China; 4 Laboratory of Applied Disaster Prevention in Water Conservation Engineering of Jilin Province, Changchun Institute of Technology, Changchun 130012, China

**Keywords:** solidified sludge, CaO, fly ash, mechanical properties, durability, microstructure

## Abstract

In order to investigate the influence of the CaO and fly ash (FA) dosage and proportion on the mechanical properties, durability, and microstructure of solidified sludge, freeze–thaw (F-T) cycles and dry–wet (D-W) cycles are conducted to study the change in appearance and the strength attenuation of CaO-FA solidified sludge. Low-field nuclear magnetic resonance (LF-NMR) is used to analyze the microstructure of the solidified sludge with various dosages and ratios of CaO-FA. The results demonstrate that the unconfined compressive strength (UCS) and direct shear strength of solidified sludge increase with the prolongation of the curing age. Furthermore, the mechanical properties of solidified sludge are improved as the ratio of CaO-FA increases. As the curing age increases, the distribution of transverse relaxation time (T_2_) becomes narrow, the spectral area decreases, and the amplitude of the LF-NMR signal shows a downward and leftward tendency. Additionally, with the increase in the number of F-T cycles and D-W cycles, the UCS of solidified sludge declines and the degree of pore deterioration increased gradually. This study offers a theoretical foundation and empirical data for the dredging and treatment of sludge in cold regions.

## 1. Introduction

Sludge is the sediment formed by physical chemistry and biochemistry in rivers. It has a high moisture content, low strength, and high compressibility, and contains high levels of pathogens, heavy metals, and other refractory organic matter [1,2]. In China, a large amount of dredged sludge is producesd in the process of dredging rivers and lakes and from the construction of ports [3,4,5,6]. According to reports, the depth of the shallow sludge in the Yitong River, Changchun city, is 0.5–1.2 m, and the dredging volume is about 1.86 million m^3^. As the long-term accumulation of this sludge will produce detrimental effects on the surrounding area [7], it is urgent to take measures to deal with it.

It is noteworthy that solidified sludge can be recycled and used in the construction of river dikes, road embankments, and lake islands or be used as a filling material for soft foundation treatments [8,9]. In this way, sludge is used as a new material for construction and is disposed of in an environmentally friendly manner.

Currently, Portland cement is widely used as a soil curing agent in practical engineering. However, the production process of this cement has obvious disadvantages, such as the consumption of non-renewable resources and energy, excessive emissions of CO_2_, and environmental pollution from waste disposal [10,11]. Therefore, there is an urgent need to develop a solidification material and technology to solidify sludge efficiently and in an ecofriendly manner.

In recent years, industrial solid waste has been used as a new material in synergistic solidification technology to solidify sludge or soil instead of traditional cement, and the application of this technology has become more and more extensive. Industrial solid waste includes CaO, FA of Class F and Class C, fibers, and so on. Nalbantoǧlu et al. [12] used FA to solidify bentonite, and their results represent an important reference for solidifying bentonite slurry. Horpibulsuk et al. [13] and Yin et al. [14] studied the strength change law of solidified soil with different moisture contents, cement contents, and other factors, and proposed a formula for the strength prediction of solidified soil with curing age. Zentar et al. [15] studied the strength, durability, and microscopic properties of siliceous–aluminous FA and cement-modified sludge, and the effects of frost damage and immersion age on the durability of solidified sludge were quantified by defining the strength loss coefficient. Takeshi et al. [16] studied the strength and durability of burnt-gypsum- and FA-cured soil. Their results showed that the strength and durability of the soil increased with the increase in the number of D-W cycles and the bassanite and FA content. MgO-FA-CO_2_ was employed as a new kind of solidification material to improve the mechanical properties of sludge [17,18,19]. The mechanical and microstructural properties of the sludge solidified by MgO-FA-CO_2_ are studied by means of durability tests. The strength and hydration properties of the solidified sludge were investigated. Moreover, the influence of CO_2_ infiltration on the mechanical properties and microstructure of MgO-FA solidified sludge was investigated. Zhu et al. [20] used calcium carbide residue as the solid alkaline activator to improve the strength of a Portland cement salified sludge. Their test results showed that the 20% cement with 20% calcium carbide residue was most effective in developing the long-term strength of the sludge.

The above curing materials and research contribute significantly to improving the properties of solidified sludge and reducing environmental pollution. Due to the large volumes of materials involved, economic cost is one of the main factors in sludge solidification. Compared with cementitious materials, FA and CaO are easily obtained and inexpensive, greatly reducing the engineering cost. The alkaline activation of FA can be used to improve the mechanical performance of sludge [21]. In addition, the major component of FA is SiO_2_ [22,23,24], and the combination of CaO and SiO_2_ can minimize the size of the micropores in the sludge [25,26]. Moreover, by using FA and CaO, carbon emissions are significantly reduced compared with using cement.

In this work, FA and CaO are used as curing agents to solidify sludge. The influence of curing agent content, the CaO/FA ratio, and curing ages on solidified dredged sludge is investigated. A series of experiments are carried out on the mechanical performance and durability of solidified sludge including UCS, direct shear tests, F-T cycles, and dry–wet cycles. LF-NMR is used to study the microstructure of the solidified sludge. The research results provide an experimental reference for the development of green and low-carbon cementitious materials and improve their application in the field of soil reinforcement.

## 2. Materials and Experiment Design

### 2.1. Materials

**Figure 1:**
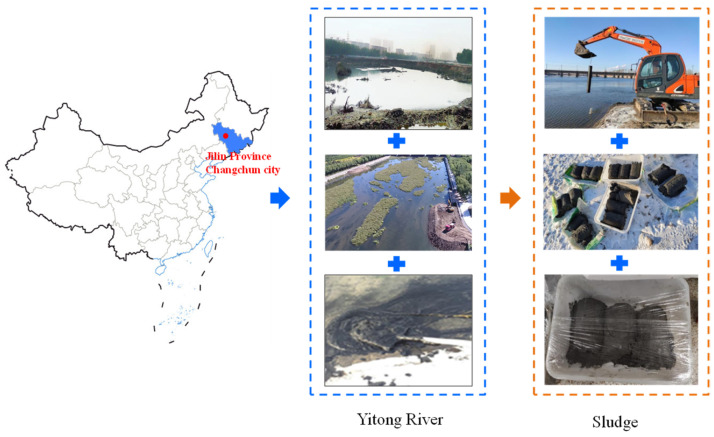
Location of the Yitong river and the procedure of in situ sampling.

The sludge used in this work was from the dredging of the Yitong river in Changchun city, China. The location of the Yitong river and the processing of in situ sampling are presented in Figure 1. The liquid limit index and plastic limit index were tested via the liquid–plastic limit combined test method, according to the Chinese specification JTG 3430-2020. The basic physical characteristics of the sludge are shown in Table 1, and the initial water content of the sludge was 29.58%. Table 2 provides the chemical composition of the FA. The density of the FA was 2.4 g/cm^3^. For the quicklime used in this work, the total mass fraction of CaO and MgO was more than 80%.

### 2.2. Preparation of Specimens

To compare and analyze the effect of the CaO/FA ratio on the performance of solidified sludge, the CaO/FA ratio was designed to be 2:8 and 4:6 (group A and group B) in this work. Each group contained three different ratios of curing agent to sludge weight, namely 10%, 15% and 20%, respectively. UCS tests were carried out after curing for 7, 14, and 28 days, respectively, with 3 parallel samples in each test. Overall, 72 solidified sludge samples were prepared. Table 3 shows the specific proportion details used in this test.

**Figure 2:**
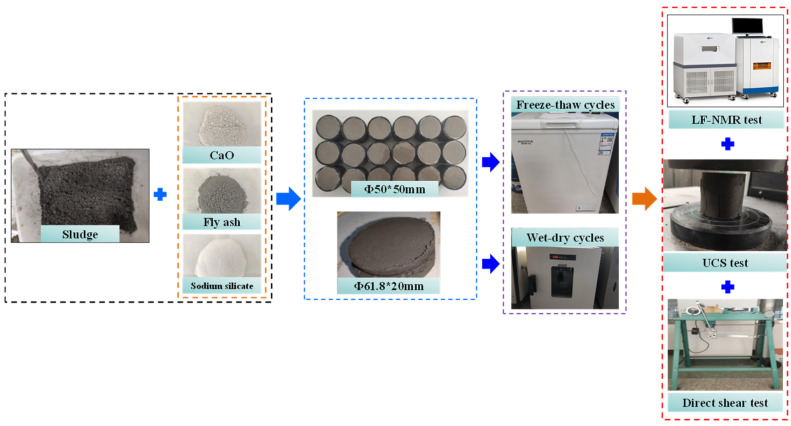
Flow chart of the testing process.

According to Chinese standard specification GB/T 50123-2019, the preparation of the specimens was divided into the following four steps: (i) we flattened and dried the sludge indoors and removed the debris, plastic, and other waste impurities from the sludge. Then, we mixed the sludge thoroughly to measure the preliminary moisture content. Subsequently, the sludge was configured with a moisture content of 40%. (ii) We weighed the sludge, CaO, and FA, respectively, and mix them fully and evenly. (iii) We manufactured 72 cylinder specimens 50 mm in diameter and 50 mm in height. (iv) We moved the specimens into the standard maintenance room for 28 days. Figure 2 shows the flow chart of the testing process and related equipment.

### 2.3. Testing Methods

#### 2.3.1. UCS Test

The UCS method used in this test was based on the Chinses national standard GB/T 50123-2019. A WDW-100 electronic universal testing machine (Kexin, Changchun, China) was used to test the mechanical properties of solidified sludge with different curing times. The loading rate was 1 mm/min until failure. This scientific instrument was manufactured in 2018. The experimental result was the average value of the three specimens.

#### 2.3.2. Direct Shear Test

Direct shear tests were conducted on the solidified sludge specimens after curing for 28 days by using a SDJ-1 electric direct shear apparatus (Nanjing Soil Instrument Factory Co., LTD, Nanjing, China) in accordance with the requirements of the Standard for Geotechnical Testing Methods (GB T 50123-2019).

#### 2.3.3. F-T Cycle

The F-T cycle test was performed with reference to ASTM D560/D560M-15. Each F-T cycle lasted 48 h and the temperature range was −20~20 °C. The specimens were frozen at −20 °C for 24 h, then were left at 20 °C for 24 h.

#### 2.3.4. W-D Cycle

The alternating W-D cycle test was performed according to ASTM D4843-1988. Each W-D cycle lasted 48 h, during which the specimens were baked in an oven at 60 °C for 24 h, then left at room temperature for 1 h, and finally placed in homemade deionized distilled water at 20 °C for 23 h.

#### 2.3.5. LF-NMR Test

To investigate the pore size distribution (PSD) inside the solidified sludge during the curing process, a MesoMR23-060H-I instrument (Niumag, Shanghai, China) was used. The equipment, with a year of manufacture of 2017, has been well maintained over the years. By using the LF-NMR technique, the effect of the curing agent and curing age on the pore water state inside the sludge could be obtained and the influence mechanism of the microstructure could be explained as well [27].

## 3. Results and Discussion

### 3.1. Strength Characteristics

**Figure 3:**
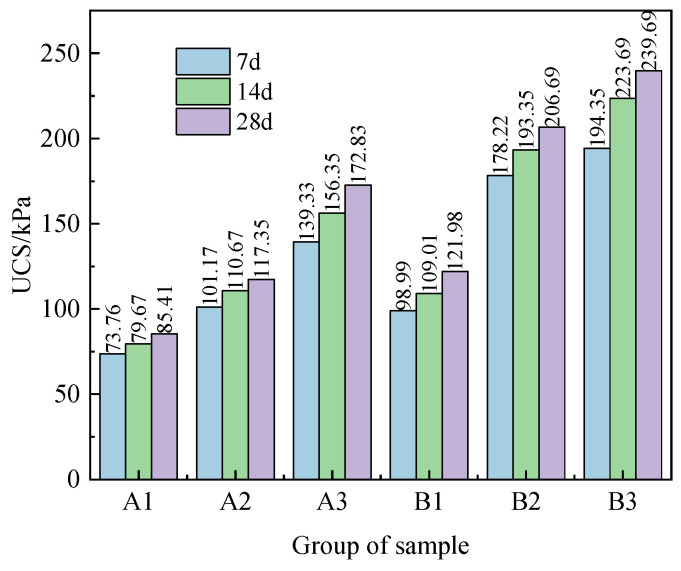
UCS of the solidified sludge specimens.

The UCS of the solidified sludge with different curing ages and CaO/FA ratios is given in Figure 3. It can be seen that the UCS of the sludge specimens gradually increases with the increase in the curing age. The strength of the specimens in each group shows the greatest growth rate at 7 d, and then the strength growth rate gradually reduces. In group B3, the strength is 194.35 kPa after 7 days of standard curing, and then increases to 223.69 kPa and 239.69 kPa at 14 d and 28 d, respectively. With the increase in curing age, the strength increases by 15.09% and 23.33%, respectively. This indicates that the hydration reaction between the solidified material and the sludge particles becomes more effective with the increase in the curing age, and the hydration products generated can fill and compact the pores between the sludge particles, which increases the bond strength between the particles.

It can also be seen from Figure 3 that the UCS increases with the increase in the amount of curing agent used under the same curing age and mixture ratio. For specimens B1, B2 and B3, after curing for 14 days, the UCS is 109.01 kPa, 193.35 kPa, and 223.69 kPa, respectively. Compared that of with B1, the UCS of B2 and B3 increases by 77.37% and 105.20%, respectively. This indicates that using more curing agent will promote the hydration reaction between CaO, FA, and pore water inside the sludge, and the hydration products generated can effectively fill the internal voids within the sludge. Thus, the mechanical properties of the sludge are improved.

Furthermore, comparing the experimental results from specimens A and B, it can be observed that the ratio of CaO to FA has a significant impact on the UCS of the solidified sludge. The UCS of A2 and B2 is 117.35 kPa and 209.69 kPa after 28 days of curing, respectively, displaying a 78.69% increase due to the increase in the CaO/FA ratio. The reason for this is that when the proportion of the FA admixture is relatively high, most of the FA adheres to the surface of sludge particles and CaO, resulting in a reduction in the early hydration reaction process. As the curing age increases, the SiO_2_ and Al_2_O_3_ in FA will further react with Ca(OH)_2_, generating C-S-H and C-A-H network structures and enhancing the strength of the cured sludge samples. Overall, the contribution of CaO to the UCS of solidified sludge specimens is higher than that of FA. Increasing the CaO/FA ratio can improve the mechanical properties of solidified sludge.

**Figure 4:**
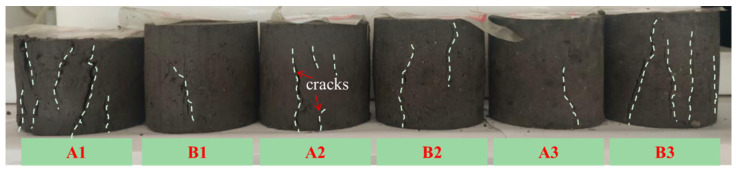
Failure mode of solidified sludge specimens.

Figure 4 illustrates that the specimens show typical ductile damage as they become thicker in the lateral direction during the loading process and that the cracks extend gradually until damaged. It can be seen that the amount of curing agent used and the CaO/FA ratio have significant effects on the failure mode of the solidified sludge. For the specimens with the same CaO/FA ratio, the number and width of cracks decrease significantly with the increase in the amount of curing agent use, while the increase in the thickness in the lateral direction decreases as well. This is because using more curing agent promotes the hydration reaction, reduces the water content, compacts the sludge, and increases inter-particle bonding. In this way, the resistance to deformation displayed by the solidified sludge increases.

### 3.2. Direct Shear Test

Shear strength is another important factor for evaluating the mechanical properties of solidified sludge. The direct shear test is carried out with different vertical pressures to determine the shear strength of solidified sludge and analyze the changes in cohesion and the internal friction angle of the solidified sludge.

**Figure 5:**
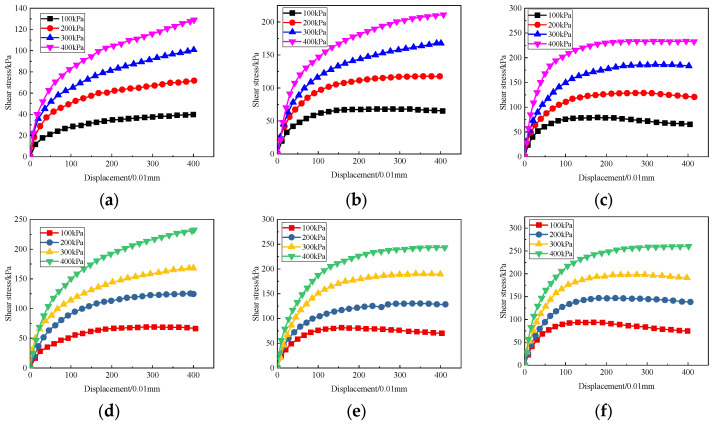
Direct shear stress–displacement curve of solidified sludge samples. (**a**) A1; (**b**) A2; (**c**) A3; (**d**) B1; (**e**) B2; (**f**) B3.

Figure 5 presents the shear stress–displacement curves of solidified sludge with different CaO/FA ratios and curing agent contents. It can be seen that the shear stress-displacement curves of different specimens have a similar trend, i.e., shear stress increasing sharply at first and then increasing slowly with the increase in displacement. This is because under the action of pressure, the arrangement of particles in the solidified sludge becomes more compact and the bonding and cohesion between particles are enhanced, indicating a typical strain hardening failure mode.

By comparing Figure 5a–c, it can be observed that under the same pressure, the shear strength of the solidified sludge increases with the increase in the amount of curing agent used. Under a pressure of 100 kPa, the shear strength of A1, A2, and A3 is 39.7 kPa, 68.42 kPa, and 79.39 kPa, respectively. Compared with A1, the shear strength of A2 and A3 increases by 1.72 times and 1.99 times, respectively. Compared with B1, the shear strength of B2 and B3 increases 1.62 times and 1.79 times, respectively.

In addition, it can be seen from Figure 5 that with the same curing agent content and pressure, increasing the CaO/FA ratio has a significant impact on the improvement in the shear strength of solidified sludge. Compared to A1, the shear strength of B1 displays an increase of 73.30%, which indicates that increasing the CaO/FA ratio improves the ability of solidified sludge to resist shear failure.

**Figure 6:**
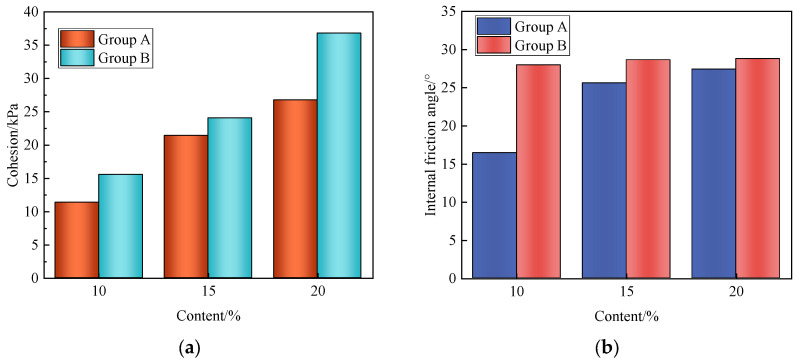
Cohesion and internal friction angle of solidified sludge. (**a**) Cohesion; (**b**) internal friction angle.

According to the Standard for Geotechnical Testing Methods (GB T 50123-2019), the cohesion and internal friction angle of solidified sludge can be obtained by using the Mohr–Coulomb law. The results are shown in Figure 6. It can be seen that both the content and proportion of the curing agent have an influence on the cohesion and internal friction angle of the solidified sludge, and the influence of the CaO/FA ratio on the cohesion is obviously higher than that on the internal friction angle.

For specimens B1, B2, and B3, the cohesion is 15.5 kPa, 24.05 kPa, and 36.80 kPa. Compared with B1, the cohesion for B2 and B3 increases by 55.16% and 137.42%, respectively. This is because the increase in the curing agent content makes the hydration reaction more effective. The pores between the sludge particles are gradually filled with hydration products. Moreover, due to the hydration reaction, the pore water content greatly reduces. In addition, the hydrated calcium silicate crystals and sludge particles will combine and form agglomerated particles with larger sizes, meaning that the friction between the particles increases and the cohesiveness is enhanced.

### 3.3. Effect of the Curing Age and Curing Agent Content on the Transverse Relaxation Time T_2_

**Figure 7:**
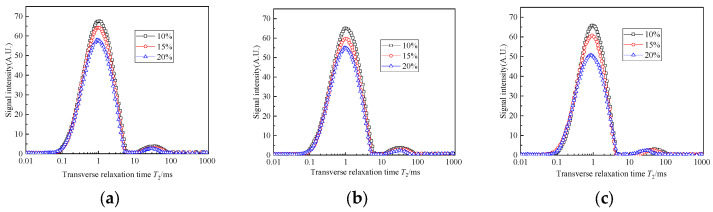
Transverse relaxation time T_2_ spectrum distribution of group A: (**a**) 7 d; (**b**) 14 d; (**c**) 28 d.

**Figure 8:**
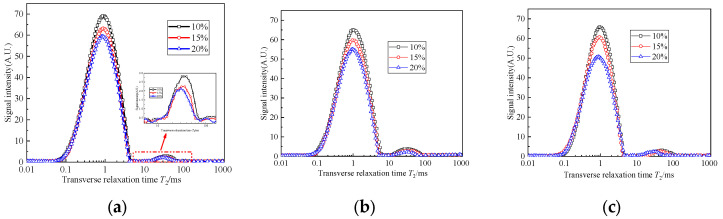
Transverse relaxation time T_2_ spectrum distribution of group B: (**a**) 7 d; (**b**) 14 d; (**c**) 28 d.

Figure 7 and Figure 8 show the curves of the transverse relaxation time T_2_ spectrum distribution of the six specimens. It can be seen that the transverse relaxation time T_2_ spectrum distribution is similar to a “double peak” distribution. The main peak range is from 0.05 ms to 5 ms, and the corresponding pore size distribution range is mainly from 0.001 μm to 0.1 μm. With the increase in curing age, the range of T_2_ distribution becomes narrower, the total spectral area decreases obviously, and the amplitude of the LF-NMR signal show a “downward” and “leftward” trend. With a higher curing agent content, the T_2_ curves of the solidified sludge at 14 d and 28 d approach each other. This is because the hydration reaction in the solidified sludge is relatively intense during the early curing age and the hydration products will fill the pores between the soil particles continuously. The pore skeleton space structure of the solidified sludge is formed. With the increase in curing age, the hydration reaction process slows down gradually, and the overall pore size distribution only changes slightly.

**Figure 9:**
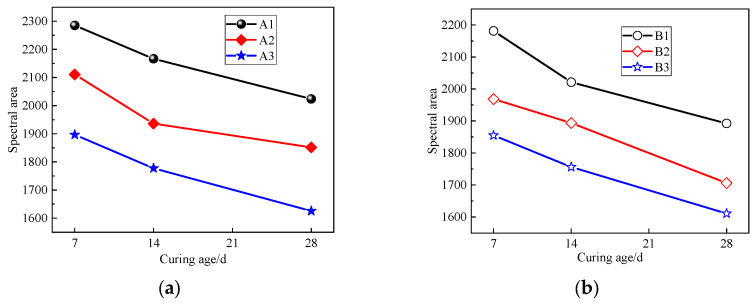
Relationship between the T_2_ spectral area of solidified sludge samples and curing age. (**a**) Group A; (**b**) group B.

As the transverse relaxation time T_2_ curve of the LF-NMR is proportional to the pore size and the internal porosity can be represented by the area enclosed by the T_2_ spectrum and the transverse coordinate, the number of internal pores in the specimen can be characterized by the peak area of the T_2_ spectrum. Figure 9 shows the area of the T_2_ spectra of specimens with different curing ages. It can be seen that the T_2_ spectral area decreases with the increase in the curing age, and the decrease in the spectral areas from 7 d to 14 d is slightly greater than that from 14 d to 28 d. This means that the number of pores inside the specimens continues to decrease with the increase in the curing age, and the hydration process is faster in the early stage. After 14 days, the hydration reaction process slows down and the T_2_ spectral area decreases gradually. In addition, using more curing agent will lead to the consumption of more free water, which leads to the smaller difference in the T_2_ spectral area between 14 d and 28 d.

The evolution mechanism of the pore structure inside the solidified sludge can be explained by the distribution of transverse relaxation time T_2_ spectra. When CaO and FA are mixed into the sludge, a series of physical and chemical reactions occur, such as hydration, ion exchange and agglomeration, calcium hydroxide crystallization, a pozzolanic effect, and so on. The cementitious hydration products C-S-H, C-A-H, and CASH with high strength and good stability are generated [28,29]. These cementitious hydration products gradually fill in the gaps between sludge particles and gradually form the spatial distribution of the pore structure. With the increase in curing age, the degree of hydration becomes more sufficient, meaning that the internal compactness of the solidified sludge increases and the cementation and cohesion between particles are enhanced. Then, the sludge is transformed from a zero-strength flow state into solidified sludge with good stability and higher strength, which agrees well with other studies [18,19,30].

## 4. Durability of Solidified Sludge

### 4.1. F-T Cycles

#### 4.1.1. Failure Characteristics after F-T Cycles

**Figure 10:**
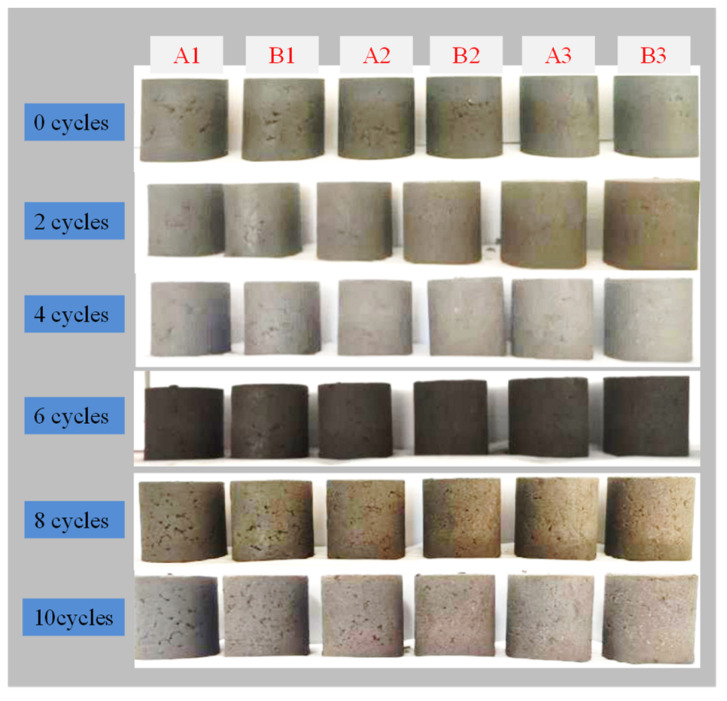
The solidified sludge samples after F-T cycles.

Figure 10 illustrates the solidified sludge specimens after F-T cycles. The surface of specimen A1 becomes rough and exhibits localized honeycomb-like pores accompanied by spalling after experiencing six F-T cycles. Similarly, A2 and A3 show these properties after 10 F-T cycles. For group B, after 10 F-T cycles, only specimen B1 becomes rough, while B2 and B3 remain unchanged. These results suggest that increasing the CaO/FA ratio can effectively enhance the F-T durability of the solidified sludge.

#### 4.1.2. UCS after F-T Cycles

**Figure 11:**
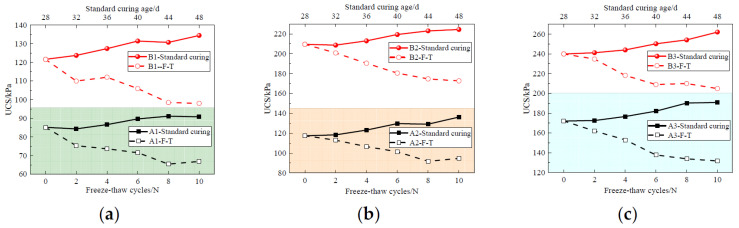
UCS of the solidified sludge samples under F-T cycles: (**a**) 10% curing agent content; (**b**) 15% curing agent content; (**c**) 20% curing agent content.

Figure 11 presents the UCS of the solidified sludge after F-T cycles. It is observed that the UCS of all the solidified sludge samples decreases with the increase in the number of F-T cycles. This is because the water inside the solidified sludge alternates between ice and water during the F-T cycles. The repeated F-T cycles destroy the continuity of the pores inside the solidified sludge, weakening the integrity and compactness of the particles. In addition, the complex chemical reactions weaken the bonds between the hydration products and the sludge particles, ultimately resulting in the continuous decline of the mechanical properties of the solidified sludge.

Moreover, it can be seen that both the content and Cao/FA ratio have a significant influence on the frost resistance of the solidified sludge. Taking group B as an example, the initial UCS of specimens B1, B2, and B3 is 121.68 kPa, 209.69 kPa, and 239.69 kPa, respectively. After 10 F-T cycles, the strength decreases to 98.01 kPa, 172.68 kPa, and 204.69 kPa, representing a reduction of 19.45%, 17.65%, and 14.60%, respectively. From Figure 11b, it can be seen that the initial UCS of A2 and B2 is 117.35 kPa and 206.69 kPa, respectively. After 10 F-T cycles, the strength of the above two specimens is 94.68 kPa and 172.68 kPa, respectively, decreasing by 19.32% and 17.65%, respectively. In this way, it can be concluded that increasing the curing agent content and CaO/FA ratio can help to improve the frost resistance and durability of CaO-FA solidified sludge.

#### 4.1.3. Evolution of Microscopic Pore Structures after F-T Cycles

**Figure 12:**
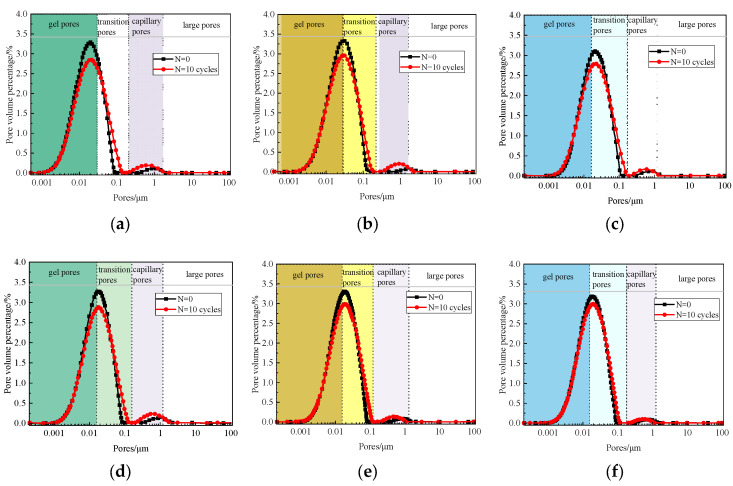
Pore size distribution of the solidified sludge samples after F-T cycles: (**a**) A1; (**b**) A2; (**c**) A3; (**d**) B1; (**e**) B2; (**f**) B3.

According to the pore size r, the pore size distribution of solidified sludge can be divided into gel pores (<0.01 μm), transition pores (0.01~0.1 μm), capillary pores (0.1~1 μm), and large pores (>1 μm) [27]. The LF-NMR is conducted by using the MesoMR23-060H-I apparatus. The pore size distribution of the specimen before and after F-T cycles is shown in Figure 12. Before F-T cycles, the major pores in the specimen are gel pores and transition pores, while the prevalence of capillary pores and large pores increases significantly after F-T cycles. The pore size distribution curves decrease significantly in the main peak signal, and the signal of the second peak shows an increasing trend. Overall, compared with group A, the changes in the pore size distribution of group B after F-T cycles are not significant, which further indicates that the F-T durability of group B is better than that of group A.

Moreover, it is noticed that the gel pores and transition pores account for 97.7% of A2’s total pore volume before F-T cycles. After 10 F-T cycles, the pore size distribution moves right and down, the proportion of gel pores and transition pores decrease to 94.1%, and the proportion of micropores and macropores increases by 2.63 times. In specimens A1 and A3, the proportion of micropores and macropores increases by 3.73 times and 3.94 times, respectively. This indicates that the F-T cycles disrupt the original pore structure of the sludge. Therefore, it can be inferred that the damage caused by F-T cycles is an irreversible cumulative deterioration of the pore structure at the microscopic level, which is consistent with the conclusions of [29,31].

### 4.2. D-W Cycles

#### 4.2.1. Failure Characteristics after D-W Cycles

**Figure 13:**
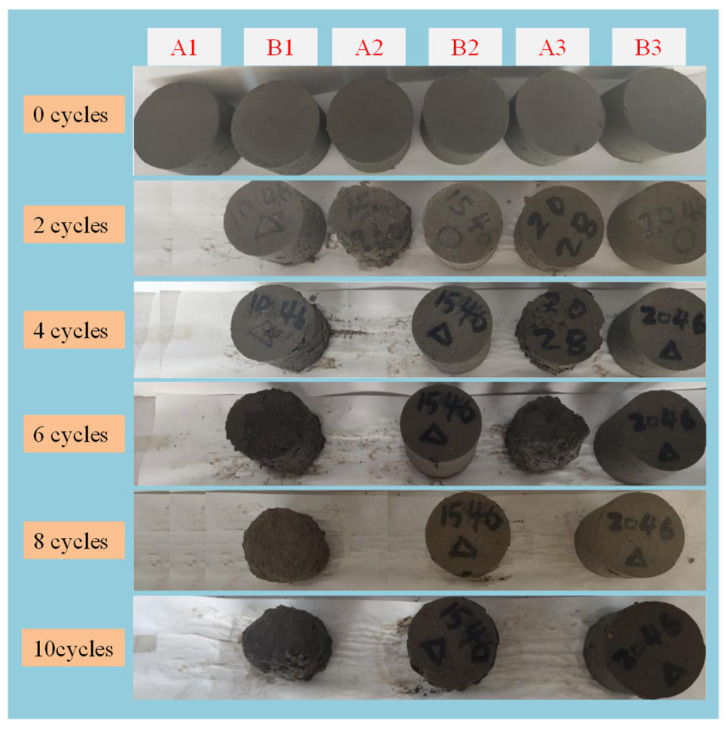
Changes in appearance of solidified sludge samples after D-W cycles.

Figure 13 shows the changes in appearance of the solidified sludge after D-W cycles. It can be seen that with the increase in the number of D-W cycles, the solidified sludge specimens crumble, detach, and disintegrate. Among all the specimens, A1 and A2 suffer the most severe damage and are completely destroyed after two D-W cycles. A3 shows extensive crumbliness and detachment on the surface after six D-W cycles. For group B, only B1 detaches obviously after 10 D-W cycles, while specimens B2 and B3 are in a good condition and have better D-W cycle resistance than the other specimens.

#### 4.2.2. UCS after D-W Cycles

**Figure 14:**
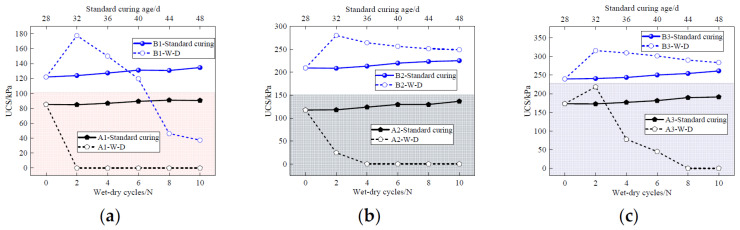
UCS of the solidified sludge samples under D-W cycles. (**a**) Curing agent content of 10%; (**b**) curing agent content of 15%; (**c**) curing agent content of 20%.

Figure 14 illustrates the UCS of solidified sludge samples after D-W cycles. Specimens not subjected D-W cycles are used as the control specimens. It can be seen that the UCS of the control specimen increases slightly with the increase in curing age. Specimen A1 loses all bearing capacity and disintegrates after one D-W cycle. The UCS of specimen A2 decreases to 24.35 kPa after two D-W cycles, which is 80% the initial strength. The UCS of specimen A3 shows a slight increase after two D-W cycles, then decreases to 45.02 kPa after six D-W cycles.

For specimens B1, B2, and B3, the UCS of the three specimens increases to 177.69 kPa, 280.03 kPa, and 315.61 kPa, respectively, after two D-W cycles. After ten D-W cycles, the UCS of B1, B2, and B3 is 37.40 kPa, 248.86 kPa, and 283.74 kPa, reducing by 78.93%, 11.17%, and 10.09%, respectively. This is because shrinkage and expansion weaken the bonds between the sludge particles and destroy the surface around the large pores, resulting in a large number of secondary pores [1,17,18]. In addition, the temperature gradient on the specimen surface under dry conditions is steep and the relative humidity under saturation is high, leading to cracking in the surface of the specimen and further resulting in an increase in the loss and absorption of water [16,19,30].

After two D-W cycles, the UCS of specimen B increases by about 31.6% to 45.5%. This is mainly due to the fact that under 60 °C dry conditions, the hydration reaction of CaO-FA is further stimulated, making the micropore structure of the solidified sludge more compact and causing the strength of the solidified sludge to increase.

#### 4.2.3. Microscope Pore Structure Distribution after D-W Cycles

**Figure 15:**
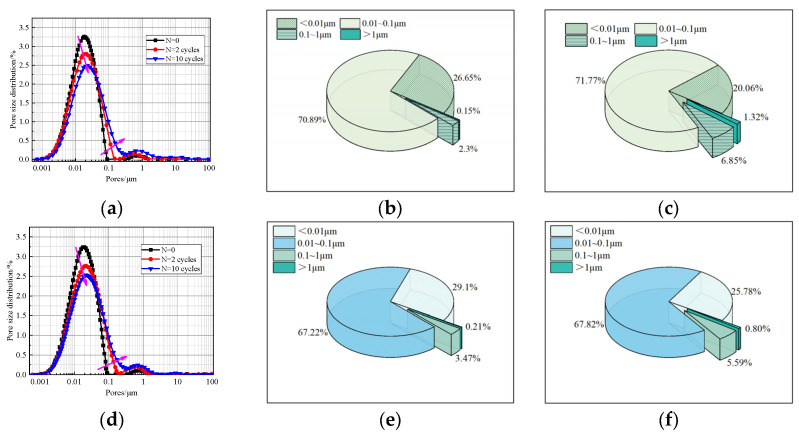
Pore size distribution of the solidified sludge samples under D-W cycles. (**a**) Pore size distribution of B2; (**b**) B2-N = 2 cycles; (**c**) B2-N = 10 cycles; (**d**) pore size distribution of B3; (**e**) B3-N = 2 cycles; (**f**) B3-N = 10 cycles.

Figure 15 displays the pore size distribution curves and pore size percentage of specimens B2 and B3 before and after the D-W cycle test. The experimental results indicate a “downward” and “rightward” trend in the first peak of the pore size distribution curve for specimens B2 and B3. In the second peak, the movement trend is “rightward”.

From Figure 15b,c, it can be seen that after two D-W cycles, the proportions of gel pores, transition pores, capillary pores, and macropores in specimen B2 are 26.65%, 70.89%, 2.3%, and 0.15%, respectively. After ten D-W cycles, the proportions of gel pores, transition pores, cap pores, and macropores are 20.06%, 71.77%, 6.85%, and 1.32%, respectively. Compared with the specimen after two D-W cycles, the proportions of capillary pores and macropores increase by 2.98 times and 8.8 times, respectively.

The variation trend in specimen B3’s pore size distribution is similar to that of specimen B2 (see Figure 15e,f). Specifically, the number of gel pores decreases significantly, while the number of capillary pores and macropores increases. It can be obtained that the D-W cycles destroy the original pore structure of the sludge and increase the connectivity between pores inside the specimen. Moreover, the proportions of capillary pores and macropores increase, and their expansion and degradation rates are significantly higher than those of gel pores and transition pores. The deterioration of the micropore structure inside the solidified sludge is a decisive factor that influences the physical and mechanical properties and durability of the sludge.

In summary, the destruction process of the solidified sludge under the action of D-W cycles can be described as follows: pore deterioration → microcracks’ initiation → surface becomes rough, and macrocracks begin to appear → surface peels off and flakes → disintegration.

## 5. Conclusions

In this work, the mechanical properties and appearance characteristics of solidified sludge with different CaO/FA ratios are compared and analyzed under different curing ages and complex environments. The variation in the internal friction angle and cohesion of solidified sludge is obtained through direct shear tests. The impact of the CaO/FA ratio and proportion on the evolution of the micropore structure in solidified sludge is analyzed systematically using LF-NMR technology. The degradation mechanisms of the mechanical properties and microstructure of solidified sludge under F-T cycles and W-D cycles are clarified. The following main conclusions are drawn:

(1) Increasing the CaO/FA ratio can significantly improve the UCS and shear strength of solidified sludge.
(2) With a higher curing agent content, the UCS and shear strength of the solidified sludge increase. Compared with a 10% curing agent content, the UCS and shear strength of specimen with a 30% curing agent content increase by 105.20% and 199%, respectively.
(3) LF-NMR can quantitatively characterize the change in micropores in the solidified sludge during curing. The transverse relaxation time T_2_ spectra become narrow and the spectra area reduces obviously with the increase in curing age. The trend of the T_2_ spectra is “downward” and “leftward”. The hydration reaction slows down and the pore skeleton space structure tends to be stable. With a higher curing agent content, the T_2_ curves at 14 d and 28 d become closer.
(4) The UCS of the solidified sludge reduces after F-T cycles or D-W cycles. Increasing the CaO/FA ratio can significantly improve the resistance of the solidified sludge to both F-T cycles and D-W cycles. Compared with the F-T cycles, the appearance damage caused by D-W cycles is worse.
(5) Periodic frozen pressure, shrinkage and wet expansion, and bulking will destroy the original pore structure of solidified sludge. The deterioration rate of large pores in the sample will be intensified, and finally the mechanical properties of the solidified sludge will continue to decline. The destruction process of solidified sludge under the action of D-W cycles can be described as follows: pore deterioration → microcracks’ initiation → surface becomes rough, and macrocracks begin to appear → surface peels off and flakes → disintegration.

## Figures and Tables

**Table 1:** Basic physical characteristics of Yitong river sludge.

pH	Density (g/cm^3^)	Initial Water Content (%)	Liquid Limit (%)	Plastic Limit (%)	Plasticity Index (%)
7.26	1.98	29.58	32.4	21.6	10.8

**Table 2:** Chemical composition of FA.

Components	SiO_2_	Al_2_O_3_	Fe_2_O_3_	CaO	MgO	K_2_O	Na_2_O	Others
Value in %	54.94	34.86	2.52	2.63	0.779	1.76	0.475	≤2

**Table 3:** Curing agent proportion of the sludge.

Group	Specimen	Curing Agent/Sludge	CaO/FA		Water Content/%	Curing Age/d
A	A1	10%	2:8	40		7, 14, 28
	A2	15%				7, 14, 28
	A3	20%				7, 14, 28
B	B1	10%	4:6			7, 14, 28
	B2	15%				7, 14, 28
	B3	20%				7, 14, 28

## Data Availability

Data is contained within the article.
